# Patterns and trends of utilization of incretin-based medicines between 2008 and 2014 in three Italian geographic areas

**DOI:** 10.1186/s12902-019-0334-y

**Published:** 2019-02-07

**Authors:** Giuseppe Roberto, Francesco Barone-Adesi, Francesco Giorgianni, Valeria Pizzimenti, Carmen Ferrajolo, Michele Tari, Claudia Bartolini, Roberto Da Cas, Marina Maggini, Stefania Spila-Alegiani, Paolo Francesconi, Gianluca Trifirò, Elisabetta Poluzzi, Fabio Baccetti, Rosa Gini

**Affiliations:** 1 Epidemiology Unit, Agenzia regionale di sanità della Toscana, Florence, Italy; 2 Department of Pharmaceutical Sciences, University of Eastern Piedmont, Novara, Italy; 3 Department of Biomedical and Dental Sciences and Morphofunctional Imaging, University of Messina, Messina, Italy; 4 Department of Medical Informatics, Erasmus University Medical Center, Rotterdam, Netherlands; 5 Department of Experimental medicine, Regional Center of Pharmacovigilance and Pharmacoepidemiology of Campania, University of Campania, Naples, Italy; 6 Local Health Unit of Caserta, Caserta, Italy; 7 National Centre for Drug Research and Evaluation, National Institute of Health, Rome, Italy; 8 Department of Medical and Surgical Science, University of Bologna, Unit of Pharmacology, Bologna, Italy; 9 Unit of DiabetologyLocal, Health Authority of North-West Tuscany, Massa, Italy

**Keywords:** Glucagon-like peptide-1 analogues, Dipeptidyl peptidase-4 inhibitors, Drug utilization, Database network

## Abstract

**Background:**

The incretin-based medicines GLP1 analogues (GLP1a) and dipeptidyl peptidase-4 inhibitors (DPP4i) are hypoglycaemic agents licensed for the treatment of type 2 diabetes mellitus (T2DM). Although these drugs possess comparable efficacy and low risk of hypoglycaemia, differences in terms of route of administration (subcutaneous versus oral), effect on body weight and gastrointestinal tolerabily can impact their actual use in clinical practice. This study aimed to describe the real-world utilization of incretin-based medicines in the Italian clinical practice.

**Methods:**

A multi-database, population-based, descriptive, cohort study was performed using administrative data collected between 2008 and 2014 from three Italian geographic areas. Subjects aged ≥18 were selected. New users were defined as those with ≥1 dispensing of GLP1a or DPP4i during the year of interest and none in the past. Trends of cumulative annual incidence of use in the general adult population were observed. New users of GLP1a or DPP4i were respectively described in terms of demographic characteristics and use of antidiabetic drugs during 1 year before and after the first incretin dispensing.

**Results:**

The overall study population included 4,943,952 subjects. A total of 7357 new users of GLP1a and 41,907 of DPP4i were identified during the study period. Incidence of use increased between 2008 (0.2‰ for both GLP1a and DPP4i) and 2011 (GLP1a = 0.6‰; DPP4i = 2.5‰) and slightly decreased thereafter. In 2014, 61% of new GLP1a users received once-daily liraglutide while 52% of new DPP4i users received metformin/DPP4i in fixed-dose. The percentage of new DPP4i users older than 65 years of age increased from 30.9 to 62.6% during the study period. Around 12% of new users had not received any antidiabetic before starting an incretin.

**Conclusions:**

During the study period, DPP4i rapidly became the most prescribed incretin-based medicine, particularly among older new user. The choice of the specific incretin-based medicine at first prescription appeared to be directed towards those with higher convenience of use (e.g. oral DPP4i rather than subcutaneous GLP1a, once-daily liraglutide rather than twice-daily exenatide). The non-negligibile use of incretin-based medicines as first-line pharmacotherapy for T2DM warrants further effectiveness and safety evaluations to better define their place in therapy.

**Electronic supplementary material:**

The online version of this article (10.1186/s12902-019-0334-y) contains supplementary material, which is available to authorized users.

## Background

Incretin-based medicines are a class of hypoglycemic agents indicated for the treatment of type 2 diabetes (T2DM) [1]. Results from clinical trials have suggested a positive risk/benefit balance for these medicines, with an hypoglycemic effect comparable to other non-insulin antidiabetic drugs (AD) and no negative effects on body weight and risk of hypoglycemia [1, 2]. The clinical efficacy of these drugs relies on the enhancement of the activity of the Glucagon-like peptide 1 (GLP1), an endogenous peptides belonging to the family of incretin hormones that exerts an important role in the glycemic homeostasis [1]. On the basis of the mechanism of action, currently available incretin-based medicines are distinguished in two main groups: GLP1 analogues (GLP1a) and dipeptidyl peptidase-4 inhibitors (DPP4i) [1, 3]. GLP1a are GLP1-receptor agonists with longer half-life compared to the naturally occurring GLP1 hormone. DPP4i, instead, can enhance the activity of the endogenous GLP1 by inhibiting its enzymatic degradation.

The mechanism of action is not the only difference between these two groups of hypoglicemic drugs. In particular, GLP1a and DPP4i respectively possess features that can differentially influence their use in clinical practice [3, 4]. For instance, GLP1a are administered subcutaneously while DPP4i are taken orally. GLP1a use can cause weight loss while DPP4i have a neutral effect. Moreover, GLP1a are generally less tolerated at gastrointestinal level than DPP4i, causing vomiting, nausea and diarrhoea.

Although these medicines have been marketed over a decade ago, little is currently known on the actual utilization of GLP1a and DPP4i in clinical practice [5–9].

n February 2008 the first incretin-based medicines (i.e. exenatide, vildagliptin and sitagliptin) received marketing authorization in Italy [5]. Since then, all licensed medicines belonging to this drug class have been reimbursed by the Italian National Health Service (NHS) as second/third line treatment for T2DM in patients with secondary failure of prior antidiabetic treatment [5, 10].

To date, only one published study provided detailed information on the utilization of these drugs in the Italian clinical practice. This study was based on the analysis of the data collected in the Monitoring Registry of the Italian Medicines Agency (AIFA) between February 2008 and August 2010 [5]. Nevertheless, due to the limited observation period, only the first three active substances available in Italy were included. Moreover, no information was provided on possible changes of utilization patterns and prescribing behaviours over time.

Indeed, for newly marketed drug classes, the number of exposed patients, as well as the characteristics of newly treated patients and the preferences of patients and prescribers with respect to specific active substances and formulations, are expected to change rapidly during the period following the introduction into clinical practice [11, 12]. In this context, evidence on the real-world utilization of medicines is paramount to understand the magnitude of possible drug-related issues, identify early signals of irrational drug use, and discuss measures and interventions to improve prescribing habits [13].

Therefore, the aim of this study was to describe the real-world patterns and trends of utilization of GLP1a and DDP4i through the analysis of routinely collected administrative data from three Italian geographic areas.

## Methods

### Data source

Italy has a tax-based, universal coverage NHS organised in three levels: national, regional, and local. Healthcare is managed to all the inhabitants by the Local Health Authority (LHA) where he/she has her regular address.

This study was based on the analysis of the administrative databases from two Italian regions in central Italy, Tuscany and Umbria, and one LHA in Southern Italy, corresponding to the province of Caserta. The three databases collect patient-leve information on the utilization of healthcare services in charge to the NHS and dispensed to all subjects who are registered with a general practitioner in the corresponding geographic areas. For each subject in the database, demographic information can be linked to all records of reimbursed drug dispensings for outpatient use. Records include information on the dispensed medicine (active substance, Anatomical Therapeutic Chemical-ATC code, brand name, formulation) as well as the date of dispensing and the number of dispensed packages.

### Study design and population

This was a multi-data base, population-based, descriptive, cohort study. Data from January 1, 2008 to December 31, 2014 were drawn from the databases of Tuscany and Caserta. As for Umbria, data were only available from January 1, 2011 up to December 31, 2014. For each year of observation, the reference study population corresponded to all subjects active into the databases at January the 1^st^. At the same date, subjects had to be ≥18 years old and have ≥365 days of look-back. Within such population, all subjects with ≥1 dispensing of any AD were identified (ATC code A10* - see Additional file 1: Appendix 1).

### Trends of prevalence and cumulative incidence of use

Users of GLP1a and DPP4i were respectively identified in each year of the study period and the annual prevalence and cumulative incidence of use were calculated. Prevalent users were subjects with ≥1 dispensing of interest during the year of reference. New users were patients with ≥1 dispensing of interest during the year of reference and none in the past. The annual prevalence of use was computed as the proportion of prevalent users in the reference population for that year. The cumulative annual incidence of use was computed dividing the number of new users in that year by the number of subjects at risk of receiving the drug of interest in the reference population (i.e. the reference population for that year minus prevalent users of the previous year). In addition, prevalence and incidence of use was also observed among AD users (i.e. ≥1 dispensing of AD). To eliminate the influence of age and sex differences across calendar years and between geographic areas, estimates of prevalence and incidence of use were standardized by age and sex using the 2012 overall study population as the reference.

### Characterization of new users

Per each year of the study period, new users of a GLP1a and DPP4i were respectively classified according to the specific active substance they started with. Moreover, newly treated patients with GLP1a and DPP4i were respectively described in terms of sex, age and AD utilization during one year before and after the first dispensing of interest (index dispensing). Prior utilization of AD was described according to the following mutually exclusive categories (see Additional file 2: Appendix 2 for the entire list of ATC codes):

1. no AD treatment;
2. insulin use, with or without non-insulin AD,
3. non-insulin AD monotherapy,
4. ≥1 non-insulin AD pharmacotherapy.

AD utilization during the year following the index dispensing was observed in patients with one complete year of follow-up (e.g. patient that died during the first 365 day of treatment were excluded from this analysis) and described according to the following non-mutually exclusive categories:

1. ≥1 additional dispensing of the index incretin (i.e. GLP1a or DPP4i),
2. ≥1 dispensing of a non-index incretin (i.e. switchers from GLP1a to DPP4i and vice versa),
3. ≥1 additional dispensing of non-incretin AD,
4. ≥1 additional dispensing of any AD,
5. Persistent use of incretins (any).

As for the latter category, patients were classified as persistent to incretin-based therapy if no treatment discontinuation was observed. Treatment discontinuation was defined as a gap >90 days between the end of the duration of a dispensing of any incretin-based medicines and the subsequent dispensing (no stockpiling was allowed) [14]. The duration of each dispensing was calculated dividing the total amount of active substance dispensed by the relevant Defined Daily Dose (DDD), which is assumed to represent the average maintenance dose per day for a drug used for its main indication in adults (https://www.whocc.no/atc_ddd_index/).

As additional analyses, characteristics of new users were further investigated within the subgroups of patients with no previous AD treatment.

### Data management and processing

Data management was performed using a distributed network approach. The infrastructure developed in the Italian national project MATRICE [15] was exploited: data from the databases of the participant institutions were exported to a common data model and managed locally using the open source software TheMatrix (http://thematrix.isti.cnr.it/). The data processing procedures is available as a Additional file 3. The resulting analytical datasets were checked by study partners and subsequently shared within the research group. All the analyses presented in this study were performed centrally at the Agenzia regionale di sanità della Toscana by using the statistical software STATA version 12.1. The data analysis procedure is available as a Additional file 3.

The use of data for the purposes of this study was approved by the relevant governance boards of each local partner.

## Results

A total of 4,943,952 adult individuals from the three geographic areas considered were included in the study. The average number of subjects per year of observation corresponded to around 4.5 million inhabitants (Table 1): 3.2 million from Tuscany, 750,000 from Caserta and 700,000 from Umbria (Additional file 4: Table S1).

**Table 1:** Description of the total study population

	2008	2009	2010	2011	2012	2013	2014
Subjects per year, *n*	3 309 447	3 444 950	3 601 468	4 506 287	4 631 043	4 705 226	4 797 459
Women, %	52.5	52.6	52.6	52.7	52.7	52.7	52.7
Subjects per age band, %							
18-44	44.8	44.0	43.1	41.6	40.8	39.8	38.9
45-64	33.0	33.0	33.2	33.3	33.3	33.3	33.3
65-84	20.7	21.1	21.3	22.0	22.4	22.9	23.2
85+	1.6	2.0	2.4	3.1	3.6	4.0	4.5
Antidiabetic users^a^ (crude), %	5.2	5.5	5.8	6.2	6.2	6.4	6.5
Antidiabetic users^a^ (age-sex standardized), %	5.6	5.8	6.1	6.3	6.2	6.2	6.2

^a^ At least one dispensing of any antidiabetic drug (ATC A10*)

**Fig. 1:**
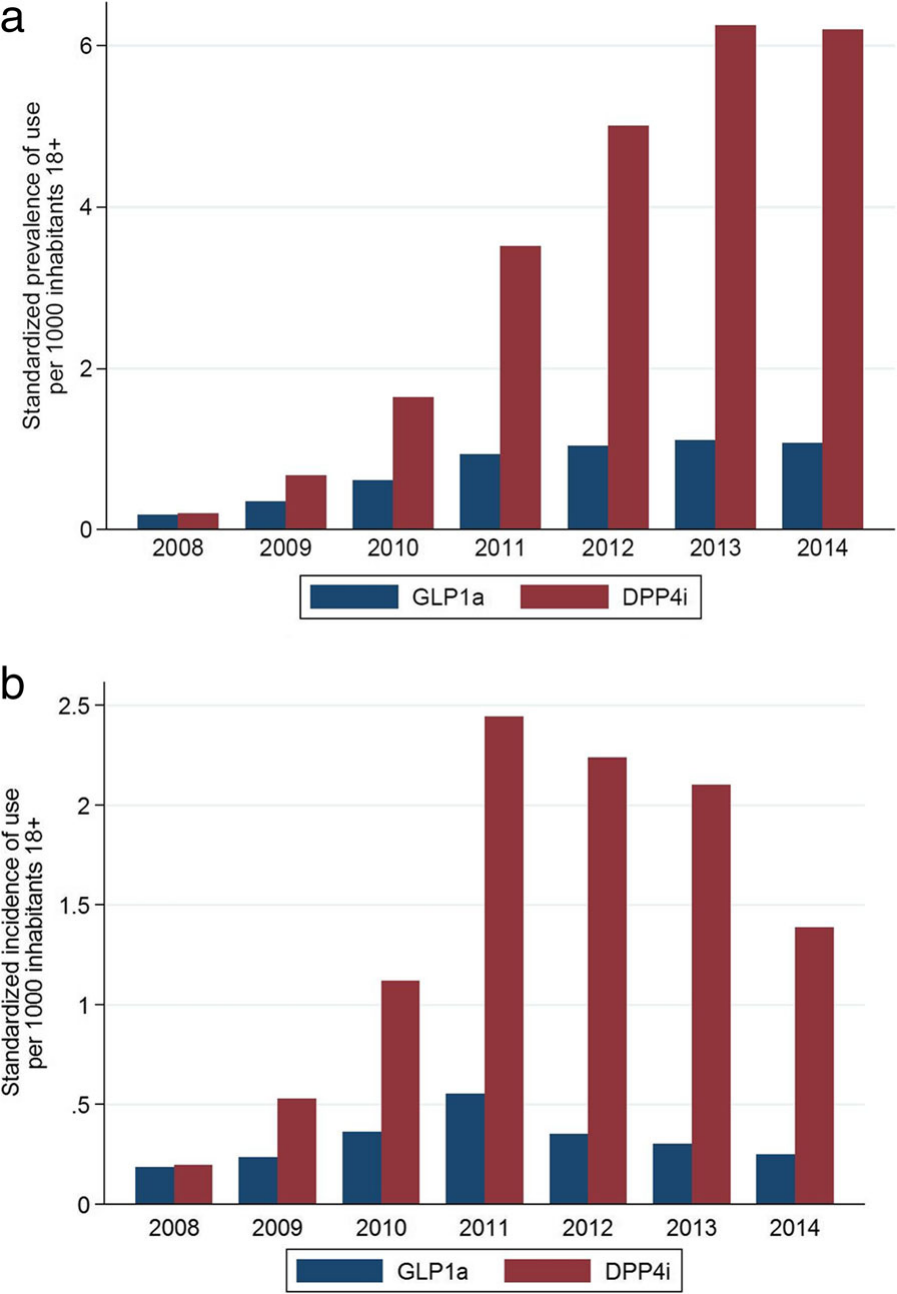
Age-sex standardized prevalence (**a**) and incidence (**b**) of use of incretin-based medicines

Overall, 7357 users of GLP1a and 41,907 users of DPP4i were identified during the study period. The prevalence of use of incretin-based medicines (Fig. 1a) increased up to 2013 (1.1‰ for GLP1a and 6.1‰ for DPP4i) and remained stable in 2014. The incidence of use in the total study population (Fig. 1b) increased between 2008 and 2011 from 0.2‰ for both GLP1a and DPP4i to 0.6‰ for GLP1a and 2.5‰ for DPP4i, and then decreased until the end of the study period (0.3‰ for GLP1a and 1.4‰ for DPP4i). Similar trends were observed when prevalence and incidence of use were estimated among AD users (Additional file 5 Figure S1 and Additional file 6 Figure S2). In particular, both in 2013 and 2014 prevalent users of GLP1a and DPP4i respectively accounted for 2 and 10% of this population. Both prevalence and incidence of use stratified by sex and age bands revealed an increasingly higher utilization of DPP4i, compared to GLP1a, in male patients with ≥65 years of age during the study period (Additional file 7: Figure S3 and Additional file 8: Figure S4).

**Fig. 2:**
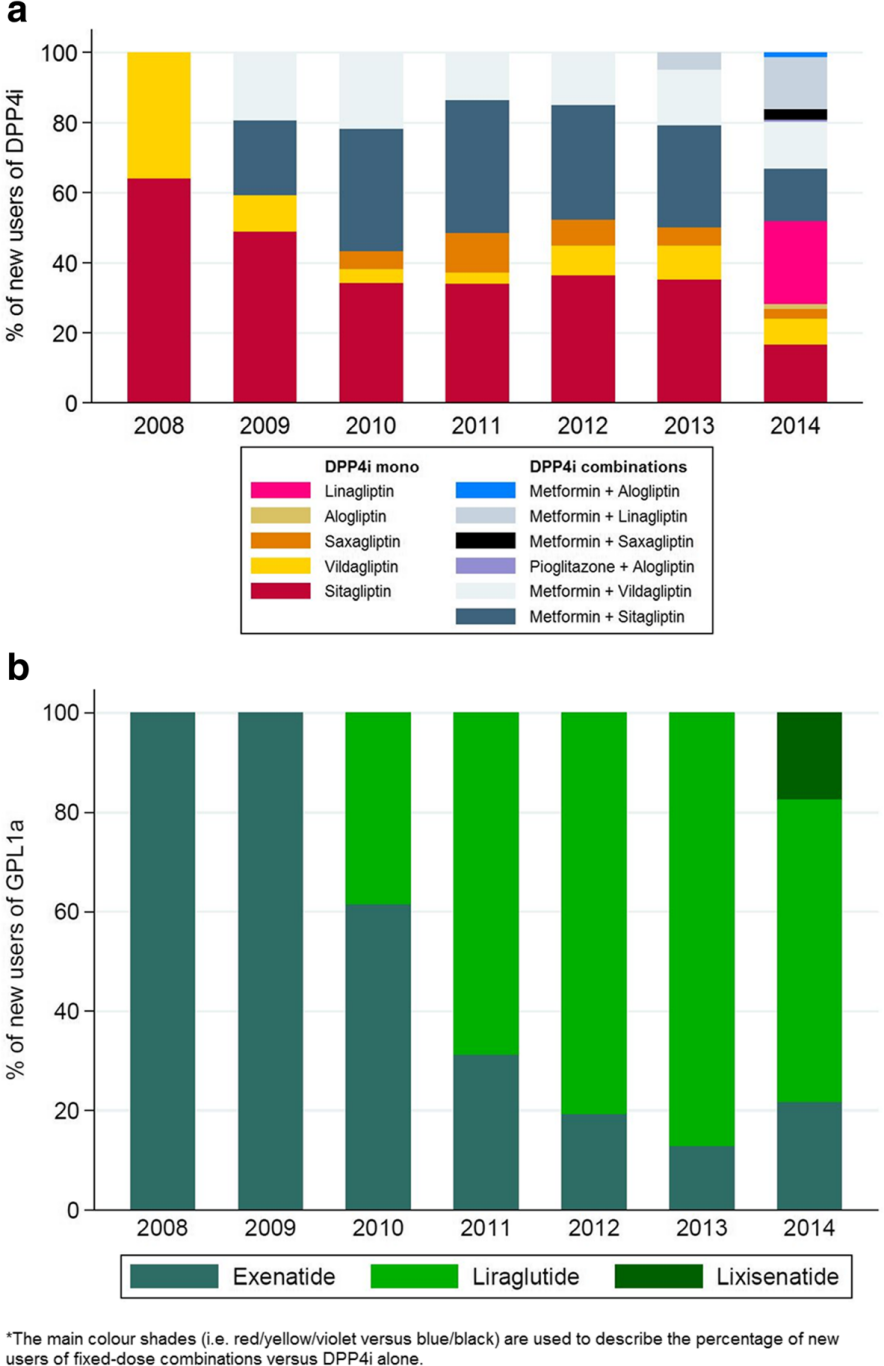
Percentage of new users of glucagon-like peptide-1 analogues (**a**) and dipeptidyl peptidase-4 inhibitors (**b**) by active

All new user of GLP1a in 2008 and 2009 received exenatide as the first prescription (Fig. 2a, data also available in Additional file 11: Table S2a). Between 2010 and 2013 new users starting with liraglutide increased from 38.5 to 87%. In 2014, 17% of new users received lixisenatide and 22% exenatide. As for first drug of choice among DDP4i (Fig. 2, panel b, data also available in table in Additional file 11: Table S2b), in 2008, 64% of patients started with sitagliptin and 36% vildagliptin. From 2009 to 2014 around 50% of all new user of a DPP4i-based therapy started a fixed-dose combination with DPP4i/metformin.

New users of GLP1a (Table 2) had a mean age of 58.1 while those starting a DPP4i (Table 3) had a mean age of 64.3. Those who received more than one non-insulin AD during the 365 days before starting incretins decreased from 58.9% in 2008 to 26.8% in 2014 for GLP1a and from 55.5 to 38.2% for DPP4i. In the whole study period, around 12% percent of new users of both GLP1a and DPP4i received no AD within 365 days preceding the index dispensing. Over the half of patients with an entire year of follow-up, corresponding to about 85% of the whole new user population, were persistent during the first year of incretin-based therapy (GLP1a = 60.6%; DPP4i = 62.4%), with a very low percentage of switchers from one to the other group (GLP1a = 5.6%; DPP4i = 1.9%).

**Table 2:** Characteristics of new users of glucagon-like peptide-1 analogues

All Data Sources		2008	2009	2010	2011	2012	2013	2014	Total
*N*		587	753	1 158	2 035	1 160	950	714	7 357
Women, %		55.2	52.7	51.5	49.3	50.3	46.5	46.2	50.0
Mean age		57.9	58.2	58.7	58.0	57.6	58.6	57.2	58.1
Age bands, *%*	18-44	8.5	9.2	8.4	10.9	10.9	11.7	12.9	10.4
	45-64	65.6	63.7	63.5	62.4	63.9	57.2	61.1	62.4
	65-84	25.9	26.8	28.0	26.1	24.8	29.3	25.9	26.7
	85+		0.3	0.2	0.6	0.3	1.9	0.1	0.5
Prior antidiabetic treatments^a^, *%*	No antidiabetics	3.7	4.8	6.6	16.5	11.6	18.6	16.9	12.3
	Insulin with/without non-insulin antidiabetics	23.0	25.4	22.3	20.6	25.7	23.5	19.0	22.6
	Non-insulin antidiabetic monotherapy	14.3	16.3	19.9	28.2	30.0	32.4	37.3	26.3
	≥1 non-insulin antidiabetic	58.9	53.5	51.2	34.7	32.7	25.5	26.8	38.9
Patients with 1 year follow-up, %		99.8	99.7	99.9	99.7	99.3	98.9	0.0	89.9
Patients with 1 year follow-up, N		586	751	1 157	2 029	1 152	939	-	6 614
Following antidiabetic treatments^b^, %	≥1 additional dispensing of a GLP1a	84.0	84.0	82.2	82.7	82.1	74.8	-	81.6
	Persistent users of incretins (any)	54.3	60.6	60.1	65.1	62.2	53.3	-	60.6
	Switchers to a DPP4i	3.4	3.7	6.2	5.7	8.1	4.7	-	5.6
	≥1 additional dispensing of non-incretin antidiabetic	91.6	91.1	86.3	86.1	84.7	79.9	-	86.1
	≥1 additional dispensing of any antidiabetic	97.1	96.7	94.5	93.2	93.1	88.2	-	93.5

*GLP1a* glucagon-like peptide-1 analogues, *DPP4i* dipeptidyl peptidase-4 inhibitors

^a^≥1 dispensing within 365 days preceding the index GLP1a dispensing

^b^Dugs dispensed during 365 days following the index GLP1a dispensing were considered

*Persistent use:* no gaps ≥90 days between the end of the duration of a dispensing and the following one

Switchers: ≥1 dispensing of a DPP4i

**Table 3:** Characteristics of new users of dipeptidyl peptidase-4 inhibitors

All Data Sources		2008	2009	2010	2011	2012	2013	2014	Total
*N*		627	1 732	3 838	10 546	9 800	9 231	6 133	41 907
Women, %		46.1	45.6	46.8	46.6	45.4	47.0	44.1	46.0
Mean age		59.2	61.5	62.0	62.6	64.5	65.9	67.4	64.3
Age bands, %	18-44	8.3	5.6	6.3	5.7	4.5	5.1	3.3	5.0
	45-64	60.8	53.8	51.2	50.2	43.6	36.8	34.2	43.8
	65-84	30.6	39.8	41.7	42.8	49.6	53.8	57.5	48.5
	85+	0.3	0.8	0.9	1.3	2.4	4.2	5.1	2.7
Prior antidiabetic treatments^a^, %	No antidiabetics	4.6	4.8	10.3	15.7	9.9	16.0	9.6	12.4
	Insulin with/without non-insulin antidiabetics	4.9	6.9	11.5	16.7	22.0	22.2	16.1	18.0
	Non-insulin antidiabetic monotherapy	34.9	30.9	26.1	27.4	29.9	28.6	36.1	29.7
	≥1 non-insulin antidiabetic	55.5	57.3	52.2	40.2	38.2	33.1	38.2	39.9
Patients with 1 year follow-up (%)		99.5	99.7	99.4	99.6	99.3	98.2	0.0	84.6
Patients with 1 year follow-up, N		624	1 727	3 815	10 504	9 731	9 067	-	35 468
Following antidiabetic treatments^b^, %	≥1 additional dispensing of a DPP4i	87.3	89.8	84.8	86.6	88.4	80.6	-	85.5
	Persistent use of incretins (any)	57.2	65.5	59.1	66.7	65.3	55.4	-	62.4
	Switcher to a GLP1a	2.9	2.5	3.5	2.3	1.6	0.8	-	1.9
	≥1 additional dispensing of non-incretin antidiabetic	92.5	81.5	74.8	76.7	76.0	72.6	-	75.8
	≥1 additional dispensing of any antidiabetic	97.0	97.3	93.0	93.4	95.3	89.9	-	93.2

*GLP1a* glucagon-like peptide-1 analogues, *DPP4i* dipeptidyl peptidase-4 inhibitors

^a^≥1 dispensing within 365 days preceding the index DPP4i dispensing

^b^Dugs dispensed during 365 days following the index DPP4i dispensing were considered

*Persistent use:* no gaps ≥90 days between the end of the duration of a dispensing and the following one

Switchers: ≥1 dispensing of a GLP1a

The additional analysis of new users with no AD dispensing during the year before the index dispensing revealed that about one out of two of these patients did not receive any other AD in the 365 days following the index dispensing and around one third were persistent incretin users.

Overall, similar trends of utilization were observed in the three geographic areas considered during the study period (Additional file 9: Figure S5 and Additional file 10: Figure S6). However, in Caserta the observed age and sex standardized incidence and prevalence of use were higher than those observed in Tuscany and Umbria, both in the whole adult population and among patients treated with any AD.

## Discussion

Through the analysis of routinely collected administrative data, this study provided evidence on the real-world utilization of incretin-based medicines in three Italian geographic areas, during a time span that covered the first 7 years since the introduction of these drugs into the Italian clinical practice.

Results from the present analysis showed a steady increase of the prevalence of use of GLP1a and DPP4i between 2008 and 2013, with a plateau in 2014. A similar figure, was reported for DPP4i in a nationwide drug utilization study performed within the whole Danish general population (i.e. no age restrictions were applied) [9]. Christensen and colleagues also reported a prevalence of use of GLP1a in 2014 that was three-fold higher than that observed in the present analysis for the same study year (3.5‰ vs. 1.1‰), although underlying differences between this and the study from Christensen et al. hamper direct comparisons (e.g. different population characteristics and standardization method, healthcare service and drug reimbursement policies).

As for the cumulative incidence of use, we observed a rapid increase up to 2011, with a trend of decrease thereafter. Other than the increasingly stringent control of the drug expenditure at national level, a possible saturation of the target population might explain such a trend, meaning that most of patients eligible to an incretin-based therapy already started the treatment during the first 4 years of utilization.

Overall, the temporal trends of prevalence and incidence of use clearly showed that DPP4i soon became the most widely used incretin-based therapy in our study population. In 2014, in fact, new DPP4i users outnumbered new GLP1a users almost 9 to one. Indeed, DPP4i allow for a less burdensome management of the disease compared to GLP1a, given the less invasive oral administration and the availability of fixed dose combination with metformin [16].

The choice of specific active substances and formulations at first prescription of a GLP1a or a DPP4i changed considerably over time and appeared to be directed towards newer medicines with a higher convenience of use. As also reported by another Danish drug utilization study [7], we observed that, during the study period, liraglutide [17] (administered once daily) almost entirely replaced exenatide [18] (administered twice daily) as the first choice for patients starting a GLP1a-based therapy (once weekly exenatide was not available in Italy during the study period). Similarly, among new users of any DPP4i-containing formulation, since 2009, when fixed dose combination with metformin became available in Italy, one out of two patients started the treatment with a formulation containing DPP4i + metformin.

The characterization of new users of incretin-based medicines showed that DPP4i treatment was increasingly started in patients ≥65 years old. Probably, the more convenient disease management associated with the use of these drugs compared to GLP1a becomes even more important for the treatment choice in the elderly. We also observed that the percentage of new users of incretin-based medicines who had already received at least two different non-insulin AD in the previous year decreased progressively during the study period, while those who were already on non-insulin AD monotherapy increased. This trend became particularly clear in both groups of new users starting from 2011. Such changes in prescribing behaviours are likely to be a consequence of the first report from the AIFA Monitoring Registry [5] on the use of incretin-based medicines in clinical practice, which was made public by the Italian Medicine Agency in January 2011. Results from the report identified baseline glycated haemoglobin as an independent predictor for effectiveness of incretin-based therapies, as also found in other observational studies [5, 19, 20]. Therefore, our findings are likely to reflect a tendency of prescribers to start incretins in subjects at an earlier disease stage and/or with a better baseline glycemic control.

The percentage of patients classified as persistent during the first year of treatment was similar in DPP4i and GLP1a users (about 60%, respectively) while the percentages of switchers from GLP1a to DPP4i and vice versa were negligible. Comparable levels of persistence were reported in literature for GLP1a and DPP4i, respectively [21, 22]. Moreover, the observed level of persistence was in line with the highest figures reported for other non-insulin ADs [23–25].

In order to promote the appropriate use incretin-based medicines, the Italian NHS allows the reimbursement of these medicines for a subset of the licensed indications only. In particular, these hypoglycemic agents can be reimbursed as second/third line treatment for T2DM when failure of previous oral hypoglycaemic pharmacotherapies is reported by prescribers [10]. In contrast with such reimbursement policy, we found that more than one in ten new users, both in the DPP4i and GLP1a group, did not receive any AD during the year preceding the first incretin prescription. Notably, while DPP4i are also licensed as a first-line treatment for T2DM in monotherapy, GLP1a are not even approved for such indication. In some cases, it is possible that generic metformin was bought without claiming reimbursement to the NHS and, thus, its dispensing was not recorded in the administrative databases. However, this is unlikely to completely explain such findings since the percentage of new incretin users without any prior dispensing of antidiabetic drug was quite stable across time, while price of copayment increased only in more recent years (http://www.regione.toscana.it/-/ticket-sui-farmaci). Moreover, the additional analyses performed within this subgroup revealed a very low level of persistence, with almost half of the patients that did not receive any other AD during the year following the first incretin prescription. Such patterns of use might be, in some cases, explained by a possible attempt to inappropriately treat prediabetes or induce weight loss [3], two conditions for which incretin-based medicines were not even approved for during the study period.This study has several strengths. First, to our knowledge this is the first, large scale, multi-database, population based study providing an overview of the utilization of GLP1a and DPP4i in the Italian clinical practice.

Second, we analyzed data from three Italian administrative databases which have been extensively used for drug utilization research studies, particularly in the contest of diabetes [6, 8, 26, 27]. Third, the study population was drawn from a source population of almost 5 million people [28] which allowed identifying and describing a very large number of incretin users. Fourth, we used a distributed network infrastructure [15], which granted consistency in the data management process across the participant units as well as compliance with privacy regulations. This study has also limitations. Although data were collected from three different Italian areas, generalizability of the results to the whole national territory cannot be assumed since drug utilization in Italy can vary significantly at regional and local level [29]. Second, the actual dose administered to patients is not recorded in Italian administrative data so that we used the DDD to estimate treatment duration. The DDD, in fact, is generally accepted as a reasonable approximation when drug utilization studies are performed in large populations of adult subjects on administrative databases. Another limitation regards the characterization of new users and is related to the administrative nature of the data source used for this study. In fact, some clinical characteristics that can influence the treatment choice in patients with T2DM are either not recorded (e.g. body mass index and glycated haemoglobin) or inaccurately identified (e.g. renal and liver disease) into Italian administrative databases.

## Conclusions

A rapid increase of the incidence of use of incretin-based medicines was observed during the first 4 years from the introduction into the Italian clinical practice. DPP4i soon became the most widely used incretin-based medicines, particularly for the elderly. The choice of specific active substances and formulations at first prescription varied over time towards newer medicines with a higher convenience of use, such as once daily liraglutide for GLP1a and fixed-dose formulations containing DPP4i/metformin. In line with current evidence and recommendations [5, 19, 20], our findings suggest that, during the study period, incretin-based medicines were increasingly started in patients at an earlier disease stage and/or with a better glycemic control. GLP1a and DPP4i users had a comparable level of persistence during the first year of treatment, while switching from GLP1a to DPP4i or vice versa was negligible. Finally, we documented the use of incretin-based medicines as first-line pharmacotherapy for type 2 diabetes in more than one out of ten new users. On the one hand, such prescribing pattern was in contrast with national reimbursement criteria and might potentially hide inappropriate utilization behaviours. On the other hand, the execution of large scale observational studies is warranted to evaluate the safety and effectiveness of incretin-based medicines as first-line pharmacotherapy for T2DM and better define the place in therapy of these hypoglycemic agents.

## Additional files

Additional file 1: **Appendix 1.** Antidiabetic drugs of interest for the study, as available in Italy during the observation period. (DOC 57 kb)

Additional file 2: **Appendix 2.** Exposure categories for the description of antidiabetic drug treatment received by new incretin users before index dispensing. (DOC 27 kb)

Additional file 3: **Appendix 3.** Data processing procedures (ZIP 43 kb)

Additional file 4: **Table S1.** Description of the total study population according to geographic areas. (DOC 79 kb)

Additional file 5: **Figure S1.** Age-sex standardized prevalence of use of incretin-based medicines among antidiabetic drug users. (PDF 3 kb)

Additional file 6: **Figure S2.** Age-sex standardized incidence of use of incretin-based medicines among antidiabetic drug users. (ZIP 4 kb)

Additional file 7: **Figure S3.** Prevalence of use of incretin-based medicines by age and gender. (ZIP 58 kb)

Additional file 8: **Figure S4.** Incidence of use of incretin-based medicines by age and gender. (ZIP 58 kb)

Additional file 9: **Figure S5.** Prevalence of use of incretin-based medicines per geographic area. (ZIP 7 kb)

Additional file 10: **Figure S6.** Incidence of use of incretin-based medicines per geographic area. (PDF 7 kb)

Additional file 11: **Table S2a** and **S2b.** Percentage of new users of GLP1 analogues by first active substance received. (DOC 83 kb)

## Abbreviations

AD: Antidiabetic drugs
ATC: Anatomical Therapeutic Chemical
DDD: Defined Daily Dose
DPP4i: Dipeptidyl Peptidase-4 inhibitors
GLP1a: Glucagone Like Peptide-1 analogues
LHA: Local Health Authority
NHS: National Health Service
T2DM: Type 2 diabetes mellitus
