# Supplementary material for: Patterns and trends of utilization of incretin-based medicines between 2008 and 2014 in three Italian geographic areas
Source: BMC Endocr Disord. 2019 Feb 7;19:18. doi: 10.1186/s12902-019-0334-y (PMC6367760; doi:10.1186/s12902-019-0334-y)
Supplement: Supplementary file 1 — Appendix 1. Antidiabetic drugs of interest for the study, as available in Italy during the observation period. (DOC 57 kb) [file 12902_2019_334_MOESM1_ESM.doc]

**Antidiabetic drugs of interest for the study, as available in Italy during the observation** period.

| **Classe farmacologica** | **ATC** | **Principio attivo** |
| --- | --- | --- |
| DDP4i | A10BH01 | sitagliptin |
|  | A10BH02 | vildagliptin |
|  | A10BH03 | saxagliptin |
|  | A10BH04 | [alogliptin](http://www.whocc.no/atc_ddd_index/?code=A10BH04&showdescription=yes) |
|  | A10BH05 | linagliptin |
|  | A10BD07 | metformin and sitagliptin |
|  | A10BD08 | metformin and vildagliptin |
|  | A10BD09 | [pioglitazone and alogliptin](http://www.whocc.no/atc_ddd_index/?code=A10BD09&showdescription=yes) |
|  | A10BD10 | metformin and saxagliptin |
|  | A10BD11 | metformin and linagliptin |
|  | A10BD13 | [metformin and alogliptin](http://www.whocc.no/atc_ddd_index/?code=A10BD13&showdescription=yes) |
| GLP1a | A10BX04 | Exenatide |
|  | A10BX07 | liraglutide |
|  | A10BX10 | Lixisenatide |
| Biguanides | A10BA01 | Fenformin |
|  | A10BA02 | Metformin |
| Sulphanylureas | A10BB01 | Glibenclamide |
|  | A10BB02 | Chlorpropamide |
|  | A10BB03 | Tolbutamide |
|  | A10BB06 | Carbutamide |
|  | A10BB07 | Glipizide |
|  | A10BB08 | Gliquidone |
|  | A10BB09 | Gliclazide |
|  | A10BB12 | Glimepiride |
| Thiazolidinediones | A10BG02 | Rosiglitazone |
|  | A10BG03 | Pioglitazone |
| Alfa glicosidase inhibitors | A10BF01 | Acarbose |
| Meglitinides | A10BX02 | [Repaglinide](http://www.whocc.no/atc_ddd_index/?code=A10BX02&showdescription=yes) |
| Insulins | A10A* | Insulin and analogues |
| Other hypoglicemic drugs in fixed combinations | A10BD01 | [phenformin and sulfonamides](http://www.whocc.no/atc_ddd_index/?code=A10BD01&showdescription=yes) |
|  | A10BD02 | [metformin and sulfonamides](http://www.whocc.no/atc_ddd_index/?code=A10BD02&showdescription=yes) |
|  | A10BD03 | [metformin and rosiglitazone](http://www.whocc.no/atc_ddd_index/?code=A10BD03&showdescription=yes) |
|  | A10BD05 | [glimepiride and pioglitazone](http://www.whocc.no/atc_ddd_index/?code=A10BD06&showdescription=yes) |
|  | A10BD14 | [metformin and repaglinide](http://www.whocc.no/atc_ddd_index/?code=A10BD14&showdescription=yes) |

GLP1a: glucagon-like peptide-1 analogues.

DPP4i: dipeptidyl peptidase-4 inhibitors.
