# Supplementary material for: Patterns and trends of utilization of incretin-based medicines between 2008 and 2014 in three Italian geographic areas
Source: BMC Endocr Disord. 2019 Feb 7;19:18. doi: 10.1186/s12902-019-0334-y (PMC6367760; doi:10.1186/s12902-019-0334-y)
Supplement: Supplementary file 2 — Appendix 2. Exposure categories for the description of antidiabetic drug treatment received by new incretin users before index dispensing. (DOC 27 kb) [file 12902_2019_334_MOESM2_ESM.doc]

Exposure categories for the description of antidiabetic drug treatment received by new incretin users before index dispensing*.

| **Exposure categories** | **Description (ATC)*** |
| --- | --- |
| Insulin with or without hypoglicemic drugs | ≥1 insulin prescription (A10A*) |
|
| Non-insulin antidiabetic monotherapy | Drugs belonging to only one of the following groups:  - Biguanides (A10BA*)  Or:  Suphanylureas or Glitinides (A10BB01, A10BB02, A10BB03, A10BB06, A10BB07, A10BB08, A10BB09, A10BB12, A10BX02)  or:  - Tiazoledindiones (A10BG*  Or:  Alfa glucosidase inhibitors (A10BF01) |
| Politherapy with hypoglycemic drugs | Drugs belonging to more than one groups among those reported above  or:  - Fixed combination (A10BD01, A10BD02, A10BD03, A10BD05, A10BD14) |

*drugs dispensed during the 365 days before index dispensing.
