# Supplementary material for: Patterns and trends of utilization of incretin-based medicines between 2008 and 2014 in three Italian geographic areas
Source: BMC Endocr Disord. 2019 Feb 7;19:18. doi: 10.1186/s12902-019-0334-y (PMC6367760; doi:10.1186/s12902-019-0334-y)
Supplement: Supplementary file 4 — Table S1. Description of the total study population according to geographic areas. (DOC 79 kb) [file 12902_2019_334_MOESM4_ESM.doc]

Table 1S. Description of the total study population according to geographic areas.

|  |  | **Tuscany** | **Umbria** | **Caserta** | **Total study population** |
| --- | --- | --- | --- | --- | --- |
| Subjects per year, n | *2008* | 2 645 787 | - | 663 660 | 3 309 447 |
|  | *2009* | 2 750 827 | - | 694 123 | 3 444 950 |
|  | *2010* | 2 867 091 | - | 734 377 | 3 601 468 |
|  | *2011* | 3 042 840 | 704 417 | 759 030 | 4 506 287 |
|  | *2012* | 3 154 644 | 703 415 | 772 984 | 4 631 043 |
|  | *2013* | 3 220 949 | 698 746 | 785 531 | 4 705 226 |
|  | *2014* | 3 287 717 | 724 636 | 785 106 | 4 797 459 |
| Women, % | *2008* | 52.7 | - | 51.8 | 52.5 |
|  | *2009* | 52.8 | - | 51.8 | 52.6 |
|  | *2010* | 52.8 | - | 51.8 | 52.6 |
|  | *2011* | 52.9 | 52.3 | 51.8 | 52.7 |
|  | *2012* | 53.0 | 52.3 | 51.8 | 52.7 |
|  | *2013* | 53.0 | 52.3 | 51.8 | 52.7 |
|  | *2014* | 53.0 | 52.4 | 51.9 | 52.7 |
| Subjects per age band, %  (18-44 / 45-64 / 65-84 / 85+) | *2008* | 42.9/33.8/21.7/1.6 | - | 52.4/29.6/16.4/1.6 | 44.8/33.0/20.7/1.6 |
|  | *2009* | 42.0/33.8/22.2/2.0 | - | 51.9/29.9/16.5/1.8 | 44.0/33.0/21.1/2.0 |
|  | *2010* | 41.0/33.9/22.6/2.5 | - | 51.2/30.5/16.3/1.9 | 43.1/33.2/21.3/2.4 |
|  | *2011* | 40.1/34.0/22.8/3.1 | 38.3/32.9/24.3/4.5 | 50.7/31.1/16.2/2.0 | 41.6/33.3/22.0/3.1 |
|  | *2012* | 39.2/33.8/23.4/3.7 | 37.6/33.2/24.5/4.7 | 49.9/31.5/16.5/2.1 | 40.8/33.3/22.4/3.6 |
|  | *2013* | 38.2/33.6/23.9/4.3 | 36.9/33.4/24.8/4.9 | 49.0/32.0/16.8/2.2 | 39.8/33.3/22.9/4.0 |
|  | *2014* | 37.2/33.5/24.4/5.0 | 37.0/33.3/24.8/5.0 | 48.0/32.5/17.2/2.3 | 38.9/33.3/23.2/4.5 |
| Antidiabetic users^1^, crude % | *2008* | 4.9 | - | 6.5 | 5.2 |
|  | *2009* | 5.2 | - | 6.6 | 5.5 |
|  | *2010* | 5.6 | - | 6.8 | 5.8 |
|  | *2011* | 5.9 | 7.1 | 6.8 | 6.2 |
|  | *2012* | 6.0 | 7.2 | 6.4 | 6.2 |
|  | *2013* | 6.1 | 7.3 | 6.5 | 6.4 |
|  | *2014* | 6.2 | 7.2 | 7.0 | 6.5 |
| Antidiabetic users^1^,  age-sex standardized % | *2008* | 5.1 | - | 8.2 | 5.6 |
|  | *2009* | 5.3 | - | 8.3 | 5.8 |
|  | *2010* | 5.6 | - | 8.5 | 6.1 |
|  | *2011* | 5.8 | 6.7 | 8.4 | 6.3 |
|  | *2012* | 5.8 | 6.7 | 7.9 | 6.2 |
|  | *2013* | 5.8 | 6.7 | 7.9 | 6.2 |
|  | *2014* | 5.8 | 6.6 | 8.3 | 6.2 |

^1^At least one dispensing of any antidiabetic drug (ATC A10*)
