# Supplementary figures and images for: Patterns and trends of utilization of incretin-based medicines between 2008 and 2014 in three Italian geographic areas
Source: BMC Endocr Disord. 2019 Feb 7;19:18. doi: 10.1186/s12902-019-0334-y (PMC6367760; doi:10.1186/s12902-019-0334-y)

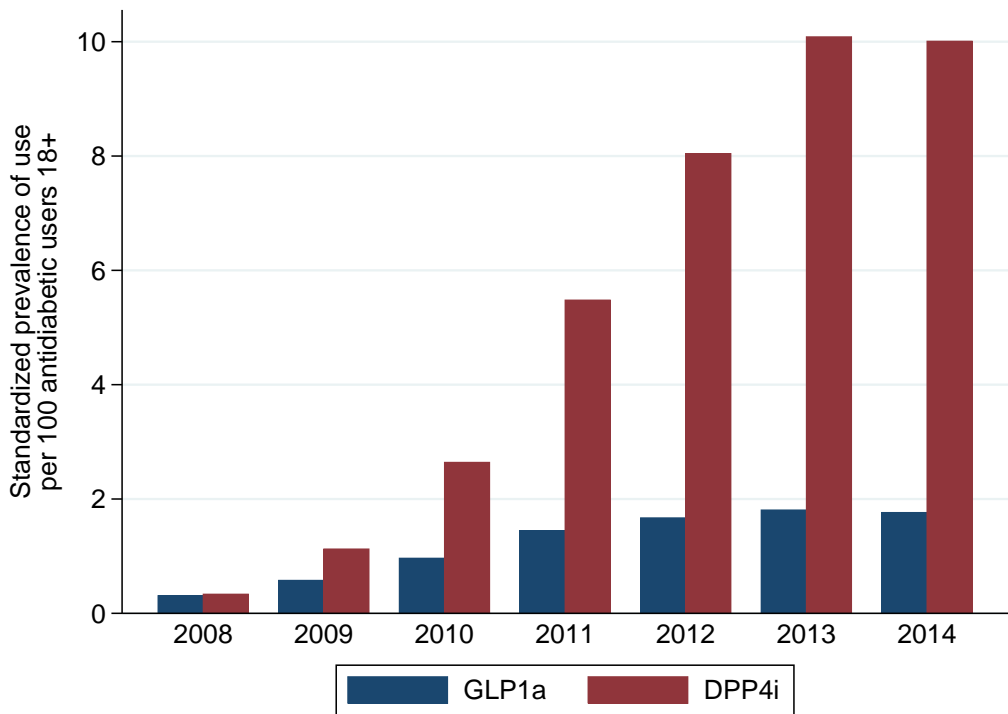

Supplement: Supplementary file 5 — Figure S1. Age-sex standardized prevalence of use of incretin-based medicines among antidiabetic drug users. (PDF 3 kb) [file 12902_2019_334_MOESM5_ESM.pdf]

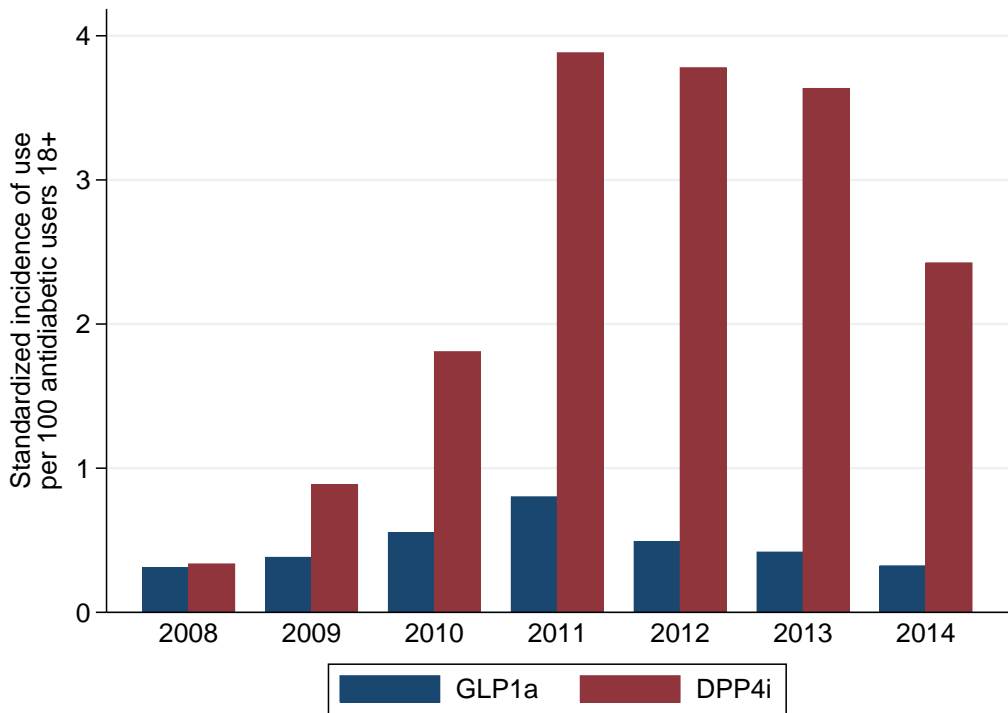

Supplement: Supplementary file 6 — Figure S2. Age-sex standardized incidence of use of incretin-based medicines among antidiabetic drug users. (ZIP 4 kb) [file 12902_2019_334_MOESM6_ESM.zip › Figure_2S.pdf]

Prevalence of use per age and gender per 1000 inhabitants 18+

18-44, F

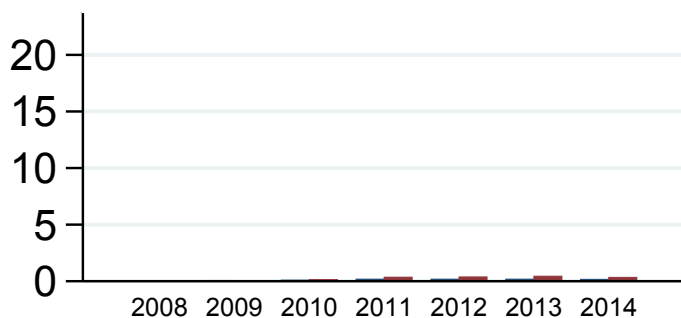

18-44, M

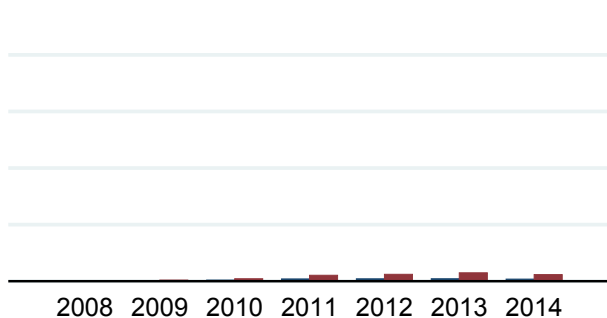

45-64, F

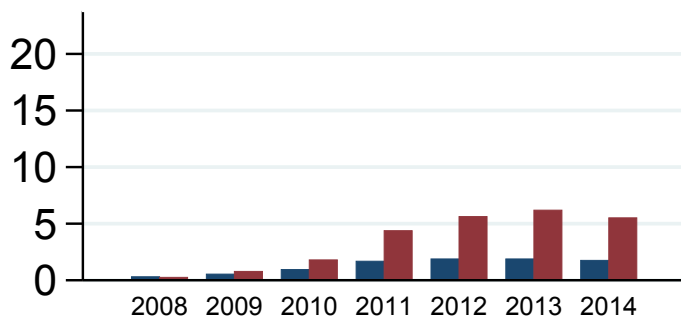

45-64, M

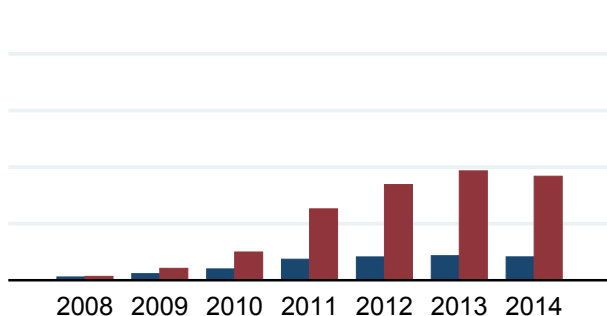

65-84, F

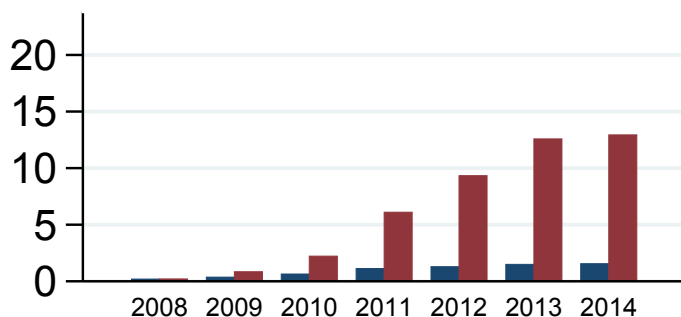

65-84, M

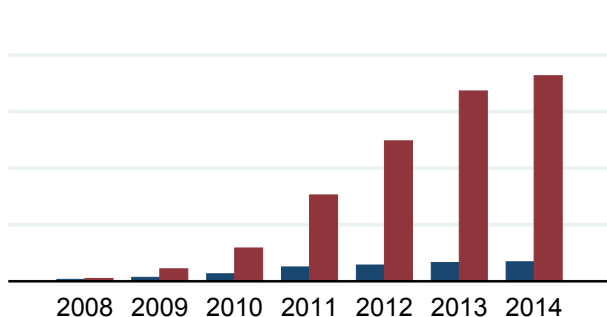

85+, F

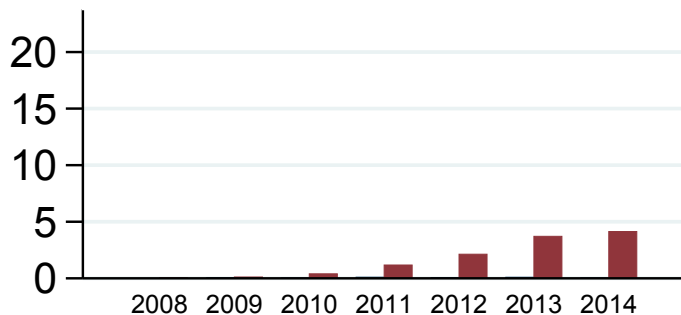

85+, M

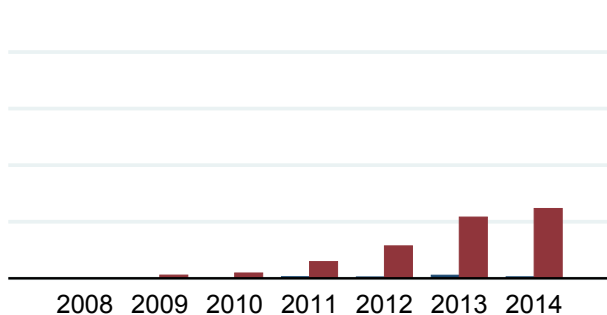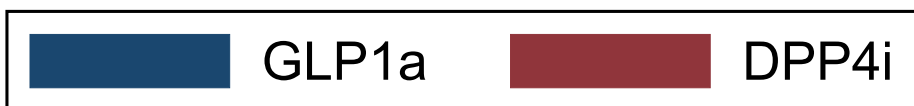

Supplement: Supplementary file 7 — Figure S3. Prevalence of use of incretin-based medicines by age and gender. (ZIP 58 kb) [file 12902_2019_334_MOESM7_ESM.zip › Figure 3S.pdf]

Incidence of use per age and gender per 1000 inhabitants 18+

18-44, F

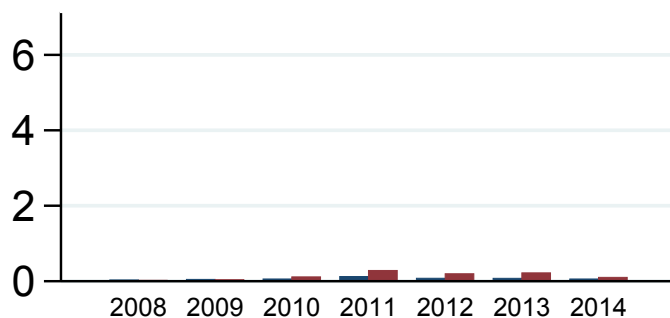

18-44, M

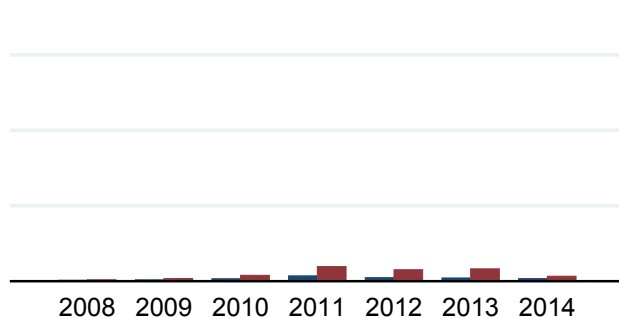

45-64, F

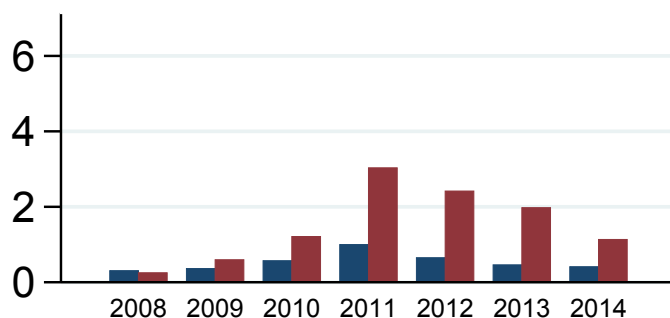

45-64, M

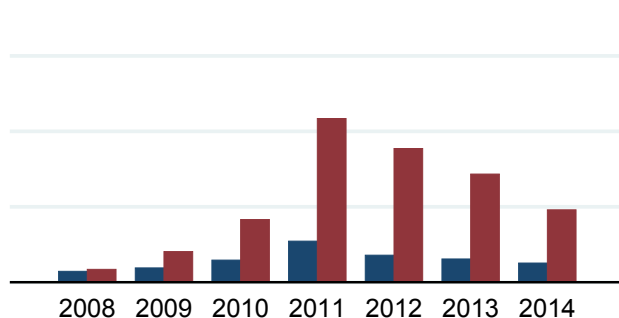

65-84, F

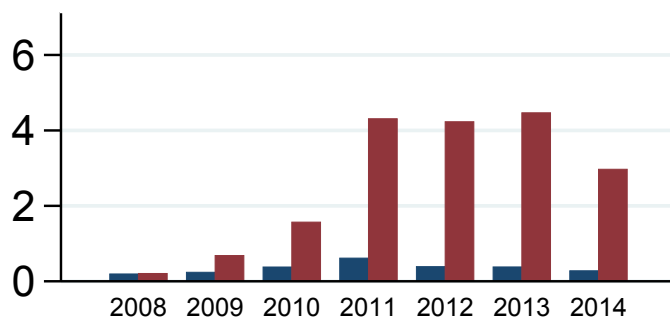

65-84, M

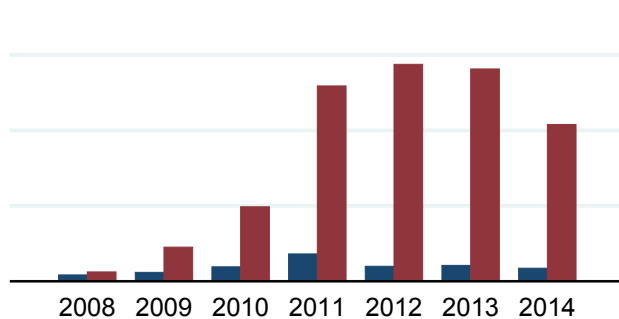

85+, F

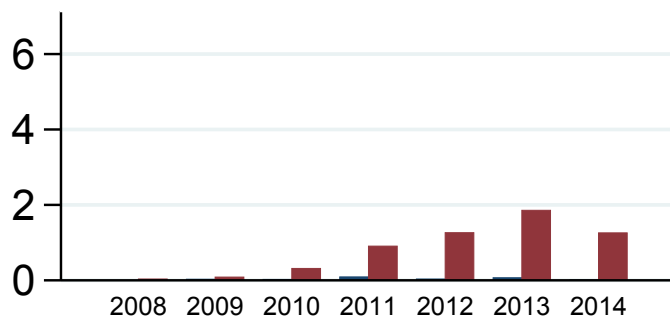

85+, M

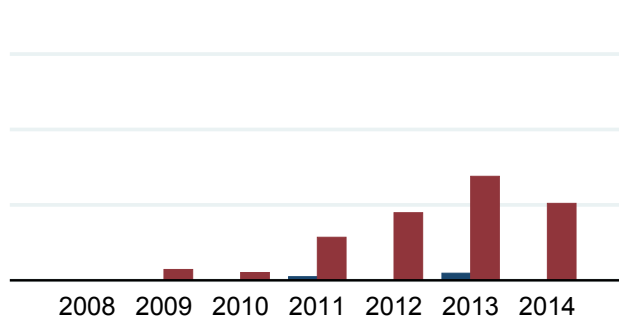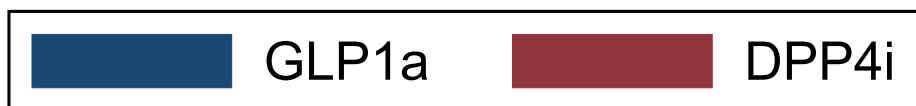

Supplement: Supplementary file 8 — Figure S4. Incidence of use of incretin-based medicines by age and gender. (ZIP 58 kb) [file 12902_2019_334_MOESM8_ESM.zip › Figure_4S.pdf]

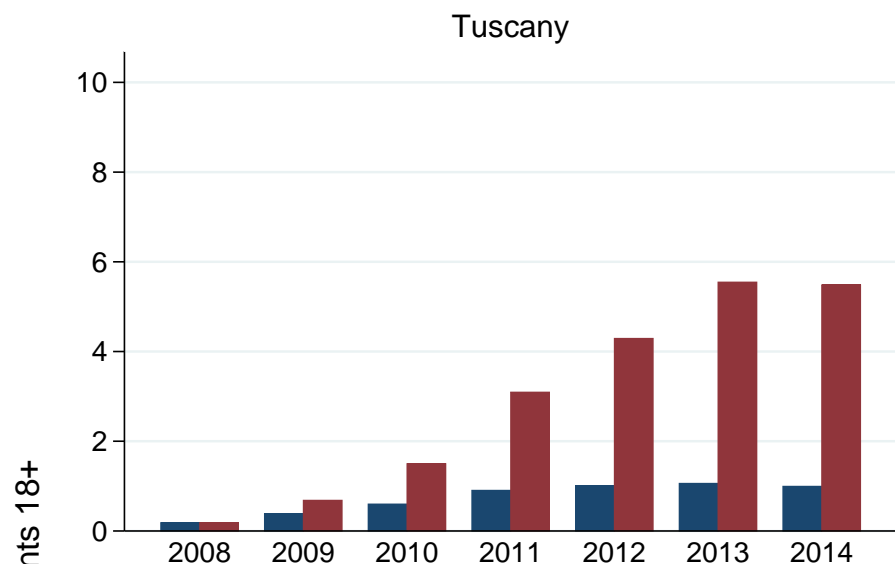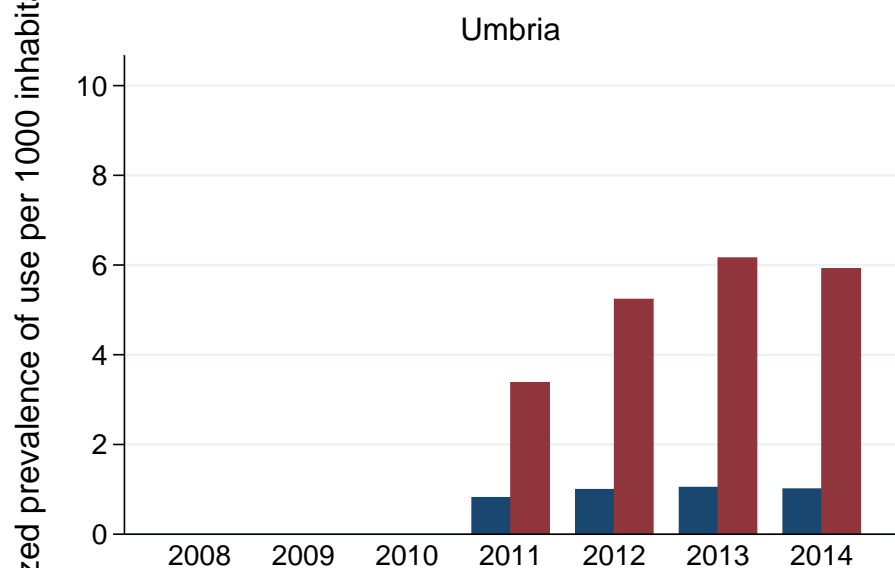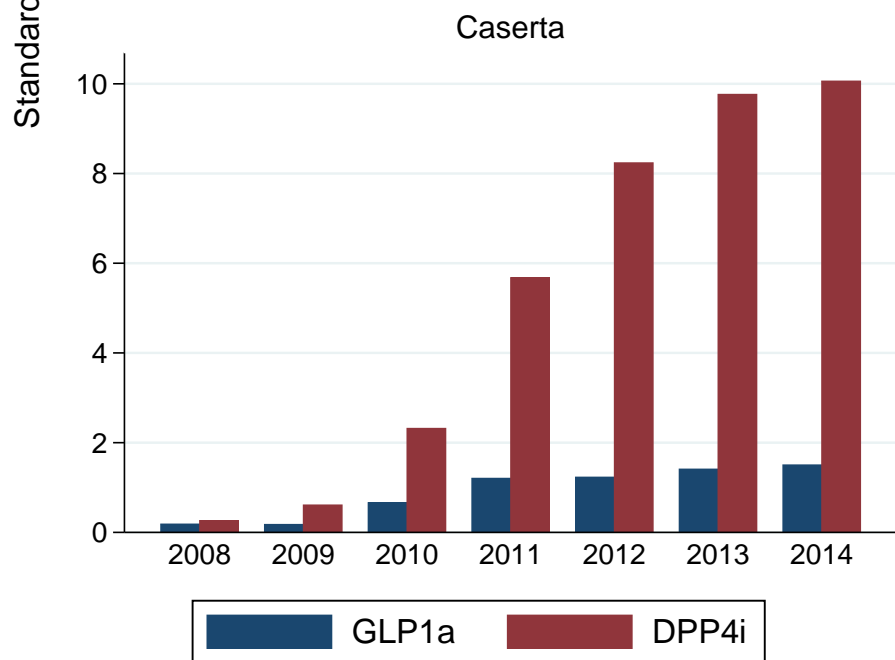

Supplement: Supplementary file 9 — Figure S5. Prevalence of use of incretin-based medicines per geographic area. (ZIP 7 kb) [file 12902_2019_334_MOESM9_ESM.zip › Figure_5S.pdf]

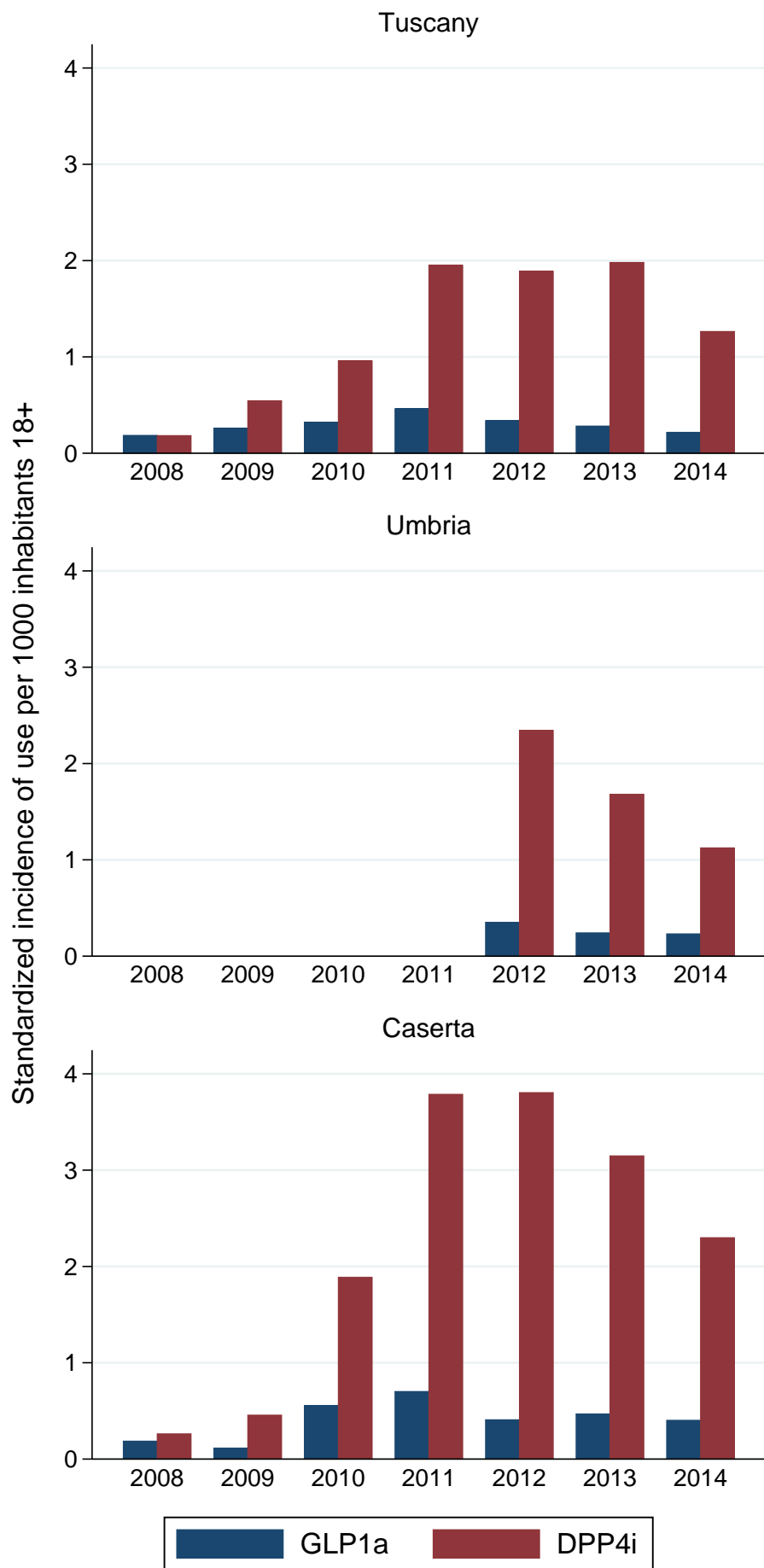

Supplement: Supplementary file 10 — Figure S6. Incidence of use of incretin-based medicines per geographic area. (PDF 7 kb) [file 12902_2019_334_MOESM10_ESM.pdf]
