# Supplementary material for: Patterns and trends of utilization of incretin-based medicines between 2008 and 2014 in three Italian geographic areas
Source: BMC Endocr Disord. 2019 Feb 7;19:18. doi: 10.1186/s12902-019-0334-y (PMC6367760; doi:10.1186/s12902-019-0334-y)
Supplement: Supplementary file 11 — Table S2a and S2b. Percentage of new users of GLP1 analogues by first active substance received. (DOC 83 kb) [file 12902_2019_334_MOESM11_ESM.doc]

**Table 2Sa. Percentage of new users of GLP1 analogues** by first active substance received.

| **Calendar year** | **New GLP1a users** | **First active substance received** | **%** |
| --- | --- | --- | --- |
| 2008 | 587 | Exenatide | 100,0 |
| 2009 | 753 | Exenatide | 100,0 |
| 2010 | 1174 | Exenatide | 61,5 |
|  |  | Liraglutide | 38,5 |
| 2011 | 2089 | Exenatide | 31,4 |
|  |  | Liraglutide | 68,6 |
| 2012 | 1216 | Exenatide | 19,5 |
|  |  | Liraglutide | 80,5 |
| 2013 | 1037 | Exenatide | 13,0 |
|  |  | Liraglutide | 87,0 |
| 2014 | 792 | Exenatide | 21,8 |
|  |  | Liraglutide | 60,9 |
|  |  | Lixisenatide | 17,3 |

**Table 2Sb. Percentage of new users of DPP4 inhibitors by first active substance received.**

| **Calendar year** | **New DPP4i users** | **First active substance received** | **%** |
| --- | --- | --- | --- |
| 2008 | 627 | Sitagliptin | 64,0 |
|  |  | Vildagliptin | 36,0 |
| 2009 | 1732 | Sitagliptin | 48,8 |
|  |  | Vildagliptin | 10,3 |
|  |  | Metformin/sitagliptin | 21,5 |
|  |  | Metformin/vildagliptin | 19,4 |
| 2010 | 3864 | Sitagliptin | 34,2 |
|  |  | Vildagliptin | 4,0 |
|  |  | Saxagliptin | 5,1 |
|  |  | Metformin/sitagliptin | 34,9 |
|  |  | Metformin/vildagliptin | 21,9 |
| 2011 | 10666 | Sitagliptin | 33,9 |
|  |  | Vildagliptin | 3,2 |
|  |  | Saxagliptin | 11,3 |
|  |  | Metformin/Sitagliptin | 38,0 |
|  |  | Metformin/Vildagliptin | 13,5 |
| 2012 | 10026 | Sitagliptin | 36,4 |
|  |  | Vildagliptin | 8,2 |
|  |  | Saxagliptin | 7,5 |
|  |  | Metformin/sitagliptin | 32,8 |
|  |  | Metformin/vildagliptin | 14,9 |
| 2013 | 9684 | Sitagliptin | 35,2 |
|  |  | Vildagliptin | 9,6 |
|  |  | Saxagliptin | 5,1 |
|  |  | Linagliptin | 0,1 |
|  |  | Metformin/sitagliptin | 29,1 |
|  |  | Metformin/vildagliptin | 15,7 |
|  |  | Metformin/linagliptin | 5,1 |
| 2014 | 6567 | Sitagliptin | 16,5 |
|  |  | Vildagliptin | 7,4 |
|  |  | Saxagliptin | 2,9 |
|  |  | Alogliptin | 1,2 |
|  |  | Linagliptin | 23,7 |
|  |  | Metformin/sitagliptin | 14,9 |
|  |  | Metformin/vildagliptin | 13,4 |
|  |  | Pioglitazone/alogliptin | 0,6 |
|  |  | Metformin/saxagliptin | 3,0 |
|  |  | Metformin/alogliptin | 14,9 |
|  |  | Metformin/sitagliptin | 1,4 |
